# Single-cell profiling uncovers extracellular vesicle-associated malignant plasma cell subpopulations driving multiple myeloma progression

**DOI:** 10.3389/fimmu.2026.1848792

**Published:** 2026-07-15

**Authors:** Lu Fan, Han Zhao, Mengya Cong, Mengxiao Zhang, Tuerxunayi Rouzi, Beibei Xie, Jifei Dai, Weiying Bao

**Affiliations:** 1 Department of Hematology, Songjiang Hospital Affiliated to Shanghai Jiao Tong University School of Medicine, Shanghai, China; 2 Shanghai Jiao Tong University School of Medicine, Shanghai, China; 3 Department of Hematology, The Second Affiliated Hospital of Anhui Medical University, Hefei, China; 4 Department of Hematology, The First Affiliated Hospital of Anhui Medical University, Hefei, Anhui, China

**Keywords:** ASS1, extracellular vesicles, ICAM, MIF, multiple myeloma, plasma cell subpopulation, scRNA-seq, tumor microenvironment

## Abstract

**Background:**

Multiple myeloma (MM) is a heterogeneous hematological malignancy characterized by the clonal proliferation of plasma cells in the bone marrow, with distinct subtypes including smoldering MM (SMM), newly diagnosed MM (NDMM), and relapsed/refractory MM (RRMM). Despite therapeutic advances, outcomes remain unsatisfactory, especially for RRMM, due to unclear heterogeneity, progression mechanisms, and crosstalk between tumor cells and the bone marrow microenvironment (BMME) via direct interactions or extracellular vesicles (EVs).

**Methods:**

Single-cell RNA sequencing (scRNA-seq) was performed on bone marrow samples from 12 MM patients, and major cell types were identified. Plasma cells were subjected to re-clustering to explore subpopulation heterogeneity. Functional enrichment analysis of differentially expressed genes (DEGs) was conducted. Cellular stemness was evaluated by CytoTRACE, and developmental trajectories were inferred via Monocle and Slingshot. Cell-cell communication was analyzed by CellChat, while transcription factor (TF) regulatory networks were identified through SCENIC analysis. Metabolic pathway activity was also assessed. Finally, loss-of-function experiments (siRNA-mediated ASS1 knockdown) were conducted to validate its functional role.

**Results:**

The analysis identified 7 major cell types. Plasma cells were further stratified into 6 subpopulations (C0-*PCSK1N*+, C1-*IGHGP*+, C2-*IGHA1*+, C3-*ASS1*+, C4-*CD27*+, C5-*STMN1*+). C3 and C5 were enriched in RRMM, while C1 and C4 were dominant in SMM. C3 exhibited hyperactive EV signature and higher stemness-like scores. Pseudotime trajectory analysis identified C3 as poorly differentiated malignant progenitors driving disease progression. C3 showed strong crosstalk with monocytes/macrophages/conventional dendritic cells (cDCs) via MIF and ICAM signaling. Key TFs regulating C3 included ATF5, TP73, MYB, CEBPB, and NFIA. Metabolic pathway analysis indicated enhanced vitamin B6 metabolism, phenylalanine metabolism, and oxidative phosphorylation in C3 and RRMM. ASS1 silencing inhibited proliferation, clonogenic capacity and migration, while promoting apoptosis in MM cells.

**Conclusion:**

Our study delineates MM heterogeneity, developmental dynamics, and regulatory networks at the single-cell level. *ASS1*+ plasma cells represent a highly malignant subpopulation associated with RRMM, driving disease progression through unique TF regulatory networks, metabolic reprogramming, and crosstalk with the BMME. These findings provide novel insights into MM pathogenesis and identify ASS1 as a potential therapeutic target for MM patients, particularly those with RRMM.

## 1 Introduction

Multiple myeloma (MM) is a malignant hematological disorder characterized by the clonal proliferation of plasma cells in the bone marrow, accounting for approximately 10% of all hematological malignancies (1, 2). Clinically, MM exhibits significant heterogeneity, with distinct subtypes including smoldering MM (SMM), newly diagnosed MM (NDMM), and relapsed/refractory MM (RRMM). SMM represents an asymptomatic precursor state with a variable risk of progression to symptomatic MM, while RRMM is associated with poor prognosis due to resistance to standard therapies. Despite advances in treatment strategies such as proteasome inhibitors, immunomodulatory drugs, monoclonal antibodies and chimeric antigen receptor (CAR) T cell immunotherapy (3–6), the clinical outcomes of MM patients remain unsatisfactory, particularly for those with RRMM.

Plasma cells, as the malignant clone in MM, play a central role in disease pathogenesis. These MM cells reside in the hypoxic bone marrow microenvironment (BMME), which triggers the release of MM-derived extracellular vesicles (EVs). In recent years, EVs are increasingly recognized as critical mediators of intercellular communication. However, conventional bulk sequencing techniques fail to capture the intrinsic cellular heterogeneity within plasma cell populations and their complex interactions with the BMME. It therefore remains poorly understood which specific plasma cell subpopulations are the main producers of these pathogenic EVs, and what transcriptional programs drive pathogenic EV biogenesis and cargo loading. Single-cell RNA sequencing (scRNA-seq) technology has emerged as a powerful tool to dissect cellular heterogeneity (7–10), identify rare cell subpopulations (11–14), and uncover dynamic molecular changes at the single-cell level (15–19). Recent studies using scRNA-seq have revealed distinct plasma cell subpopulations with unique gene expression profiles in MM (20), which may be associated with disease progression and treatment response. Nevertheless, the transcriptional characteristics, developmental trajectories, and functional roles of plasma cell subpopulations across different MM subtypes (SMM, NDMM, and RRMM) remain poorly defined.

Moreover, the BMME, consisting of monocytes/macrophages, T/NK cells, dendritic cells, and stromal cells, plays a crucial role in supporting MM cell survival, proliferation, and drug resistance through complex cell-cell communication networks. Dissecting the intercellular crosstalk between plasma cells and other cell types in the BMME is essential for understanding MM pathogenesis and identifying potential therapeutic targets. Additionally, transcription factors (TFs) and metabolic pathways are key regulators of MM cell biology, and their dysregulation may contribute to disease progression and subtype-specific characteristics. However, the TF regulatory networks and metabolic rewiring in distinct plasma cell subpopulations across MM subtypes have not been systematically investigated.

In this study, we utilized scRNA-seq data from the Gene Expression Omnibus (GEO) database (accession number GSE223060) to comprehensively analyze the cellular heterogeneity, developmental trajectories, cell-cell communication patterns, TF regulatory networks, and metabolic characteristics of bone marrow cells from MM patients. Our objectives were to: (1) identify distinct plasma cell subpopulations and their distribution across SMM, NDMM, and RRMM; (2) delineate the developmental trajectory of plasma cell subpopulations and their role in disease progression; (3) explore the intercellular communication networks between plasma cells and other cell types in the BMME; (4) uncover the TF regulatory modules and metabolic pathways specific to plasma cell subpopulations and MM subtypes. The findings of this study provide novel insights into MM pathogenesis and may facilitate the development of personalized therapeutic strategies for MM patients.

## 2 Materials and methods

### 2.1 Data acquisition and processing

All scRNA-seq data were downloaded from the GEO database (https://www.ncbi.nlm.nih.gov/geo/) with the accession number GSE223060. The dataset employed for single-cell analysis comprised bone marrow mononuclear cell (BMMC) samples collected from MM patients. The data were derived from publicly available databases, so ethical review was unnecessary for this study.

### 2.2 Single-cell data processing

Raw gene expression data were imported into R (v4.4.1) for downstream analysis using the Seurat package (v4.3.0) (21–23). Quality control (QC) protocols were carried out to eliminate low-quality cells and doublets: doublet detection was accomplished using the DoubletFinder package (v2.0.3), and cells were filtered to meet the following QC criteria: (1) the number of detected genes (nFeature) ranged from 300 to 6,000; (2) total UMI counts (nCount) were restricted to 500-100,000; (3) the proportion of mitochondrial gene expression relative to total cellular UMI counts was less than or equal to 25%; and (4) the proportion of erythroid gene expression relative to total cellular UMI counts did not surpass 5%.

Subsequently, the gene expression matrix of filtered cells underwent logarithmic normalization and linear regression analyses, by leveraging the “NormalizeData” and “ScaleData” functions incorporated in the Seurat R package (v4.3.0). Specifically, logarithmic normalization was performed using the “NormalizeData” function (24). Next, the “FindVariableFeatures” function was utilized to identify the top 2,000 highly variable genes (HVGs), and these HVGs were further standardized via the “ScaleData” function. Batch effects were corrected via the Harmony package (v0.1.1), and the first 30 principal components (PCs) were retained for subsequent downstream analyses.

### 2.3 Cell type identification and Ro/e analysis

The initial 30 PCs were chosen for dimensionality reduction and clustering analyses. After completing dimensionality reduction and clustering procedures, the resulting data were mapped onto a two-dimensional (2D) space through the Uniform Manifold Approximation and Projection (UMAP) algorithm, which facilitated the identification of cell types (25–27). Markers specific to each cell type were obtained from the CellMarker database (http://xteam.xbio.top/CellMarker/) and verified by cross-referencing with relevant literature. These cell type-specific markers were then employed for the annotation of cell clusters, which allowed for the identification of distinct cell populations as well as the characterization of their distribution patterns and relative proportions. For a more in-depth investigation of the intrinsic heterogeneity within plasma cells, the plasma cell population was subjected to re-clustering, and each resulting subcluster was annotated based on its unique gene expression profile. Next, we calculated Ro/e for each cell type in different disease subtypes to quantify the preference of each cell type. Ro/e is the ratio of observed cell number over the expected cell number (Ro/e = observed/expected) (28). The chi-squared test was used to determine the predicted cell number for each combination of cell types and disease subtypes. One cell type was identified as being enriched in a specific disease subtype if Ro/e > 1.

### 2.4 Enrichment analysis of differential gene expression

Differentially expressed genes (DEGs) corresponding to each cell type were identified via the “FindAllMarkers” function in the Seurat package, utilizing the Wilcoxon rank-sum test with its default parameter settings. Stringent filtering criteria were applied to screen DEGs: a log2 fold change (LogFC) exceeding 0.25 and expression in no less than 25% of cells within the respective cell cluster. To clarify the functional significance of these identified DEGs, functional enrichment analyses were conducted using the clusterProfiler (v4.6.2) and SCP (v0.4.8) packages, with gene sets derived from the Gene Ontology (GO) database (29, 30) and the Molecular Signatures Database (MSigDB), which was accessed through Gene Set Enrichment Analysis (GSEA) (31, 32).

### 2.5 AUCell score

Quantification of signature activity scores was performed with the scgmt package (v0.0.3; https://github.com/ZhaoLabs-SJTU/scgmt) (33, 34), where the “AUCell” approach was designated through the “method” parameter. As a widely recognized algorithm for quantifying gene set activity in scRNA-seq data, AUCell accepts a pre-defined gene set as input and produces a quantitative “activity score” that reflects the functional activity of the target gene set in each individual cell.

### 2.6 Developmental trajectory inference

Cellular stemness potential was assessed via the CytoTRACE (v0.3.3) (35, 36), with the findings of this assessment delineating the temporal dynamics of cellular differentiation.

Cell differentiation trajectories were established with the Monocle package (v2.24.0) (37–39). Dimensionality reduction was achieved through the UMAP algorithm, after which trajectory visualization was conducted using the “plot_cell_trajectory” function. Subsequently, cell subpopulations were ordered according to the pseudotime. Genes exhibiting synchronized expression alterations along the pseudotime trajectory were identified, and their expression patterns were visualized as a pseudotime heatmap.

The Slingshot package (v2.6.0) (20, 40) was utilized for the inference of cell lineages and pseudotime. This tool employs a clustering-derived minimum spanning tree to outline lineage structures and fits branch curves to these lineages by means of simultaneous principal curves. The “getCurves” function was applied to obtain smoothed trajectory curves (41).

### 2.7 Cell-cell communication

Putative intercellular crosstalk between distinct cell populations was inferred via the CellChat package (v1.6.1) (42, 43), where a significance cutoff was established at a P-value of 0.05. Specifically, we used the “identifyCommunicationPatterns” function to quantify the number of intercellular communication patterns, and applied the “netVisual_diffInteraction” function to visualize the differences in cellular communication strength (44).

### 2.8 Single-cell regulatory network inference and clustering analysis

For the identification of the most notably dysregulated TFs in each cell subpopulation, single-cell regulatory network inference and clustering analyses were conducted with the SCENIC package (v1.3.1) implemented in Python software (v3.7) (45–47). Specifically, the GRNBoost algorithm was employed to predict potential target genes corresponding to each TF. Following this, DNA motif analysis was carried out to pinpoint direct binding targets of these TFs. Finally, the activity of regulons in individual cells was measured using the AUCell approach, and the top 5 TFs with the highest regulon activity scores were identified as key candidates.

### 2.9 Cell lines and culture conditions

Human MM cell lines RPMI 8226 and U266 were obtained from the American Type Culture Collection (ATCC). Cells were authenticated by short tandem repeat (STR) profiling and routinely tested negative for mycoplasma contamination. Both cell lines were maintained in RPMI-1640 medium (Gibco, Thermo Fisher Scientific) supplemented with 10% fetal bovine serum (FBS; Gibco) and 1% penicillin-streptomycin (Gibco). Cells were cultured at 37 °C in a humidified incubator with 5% CO_2_ and passaged every 2–3 days to maintain logarithmic growth.

### 2.10 siRNA-mediated knockdown of ASS1

Two independent siRNAs targeting human ASS1 (si-ASS1#1 and si-ASS1#2) and a non-targeting control siRNA (si-Ctrl) were synthesized by GenePharma (Shanghai, China). Cells were transfected with siRNAs using Lipofectamine™ RNAiMAX Transfection Reagent (Invitrogen, Thermo Fisher Scientific). The detailed sequences of the siRNAs are provided in **Supplementary Table 1**.

### 2.11 RNA extraction and quantitative real-time PCR

Total RNA was extracted 48 h post-transfection using TRIzol™ Reagent (Invitrogen) according to the manufacturer’s protocol (48). cDNA was synthesized via PrimeScript™ RT Reagent Kit (Takara). qRT-PCR was conducted with SYBR™ Green Master Mix (Applied Biosystems, Thermo Fisher Scientific) on a QuantStudio™ 6 Flex Real-Time PCR System (Applied Biosystems), and mRNA expression levels were calculated by 2^-ΔΔCt^ method (GAPDH as internal reference) (49–51), with 3 technical and 3 biological replicates. The primer sequences are listed in **Supplementary Table 1**.

### 2.12 Colony formation assay

After transfection, cells were seeded in 6-well plates and cultured for 10-14 days. Colonies were fixed and stained with 0.1% crystal violet (Solarbio), followed by the counting analysis using a light microscope and ImageJ software (NIH).

### 2.13 Cell viability assay

According to the manufacturer’s protocol, cell viability was determined by Cell Counting Kit (CCK) -8 (Dojindo Laboratories) (52). 10 μL CCK-8 reagent was added and incubated at 37 °C for 2 h. The absorbance at 450 nm was detected with a microplate reader (BioTek Synergy H1).

### 2.14 Transwell migration assay

Cell migration was detected by transwell chambers with pore polycarbonate membranes (Corning) (53–55). Cells in serum-free RPMI-1640 medium were added to the upper chamber, while RPMI-1640 with FBS was added to the lower chamber. After incubation at 37°C, the migrated cells in the lower chamber were stained and counted.

### 2.15 Apoptosis analysis by flow cytometry

Apoptosis was assessed using an Annexin V-FITC/PI Apoptosis Detection Kit (BD Biosciences) (56). Samples were analyzed using a BD FACSCanto™ II flow cytometer (BD Biosciences). Data were processed with FlowJo software. Early apoptotic cells were defined as Annexin V^+^/PI^-^, and late apoptotic cells as Annexin V^+^/PI^+^. Total apoptosis rate was calculated as the sum of early and late apoptotic fractions.

### 2.16 Data statistics

The Kolmogorov-Smirnov (KS) test was relied on for the GSEA (57, 58). In the CellChat analysis, permutation tests were utilized to detect significant ligand-receptor (L-R) interactions. For CytoTRACE, the Wilcoxon rank-sum test was employed for the comparison of scores among different groups. In Monocle, generalized linear modeling (GLM) was applied to pinpoint genes that exhibited temporal expression variations across the pseudotime trajectory. Context-dependent statistical tests were applied to the analysis of TFs, with the goal of identifying cell population-specific regulons.

All experiments were independently performed at least 3 times. All values are presented as the mean ± standard deviation (SD). Statistical analysis was conducted using GraphPad Prism 9.0. Differences between two groups were analyzed using unpaired Student’s t-test, and comparisons among multiple groups were performed using one-way ANOVA followed by Tukey’s *post hoc* test. A two-sided P < 0.05 was considered statistically significant.

## 3 Results

### 3.1 Cell heterogeneity in SMM, NDMM, and RRMM

**Figure 1:**
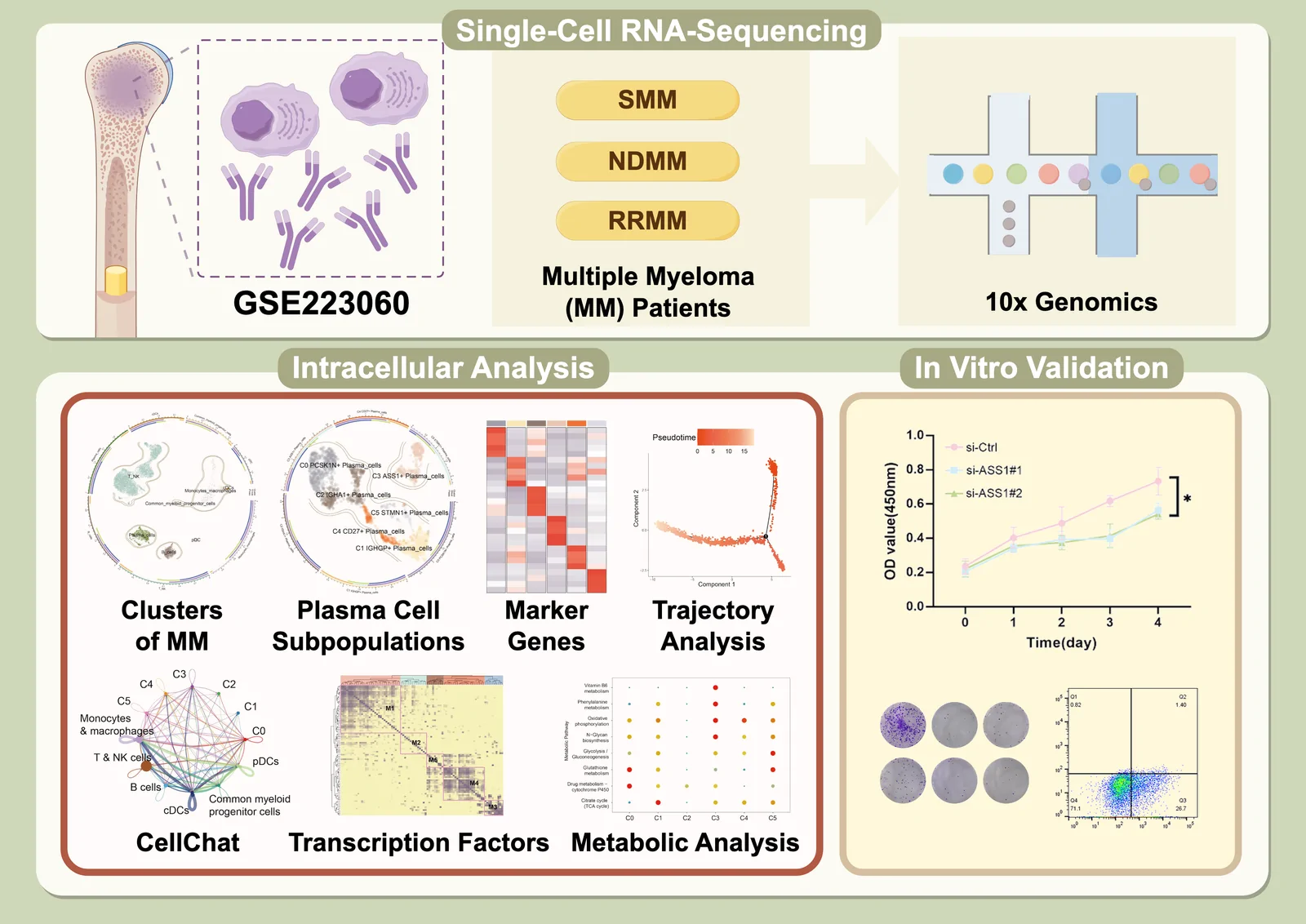
Graphical abstract. The analysis workflow of this study. We conducted single-cell sequencing analysis on the GSE223060 dataset to identify distinct plasma cell subpopulations. Subsequently, through a series of bioinformatic analyses including enrichment analysis, pseudotime trajectory analysis, intercellular communication analysis, transcription factor regulatory analysis, and metabolic pathway analysis, we elucidated the functional significance of these plasma cell subpopulations in SMM, NDMM and RRMM. Then, this was further validated through *in vitro* experiments.

**Figure 2:**
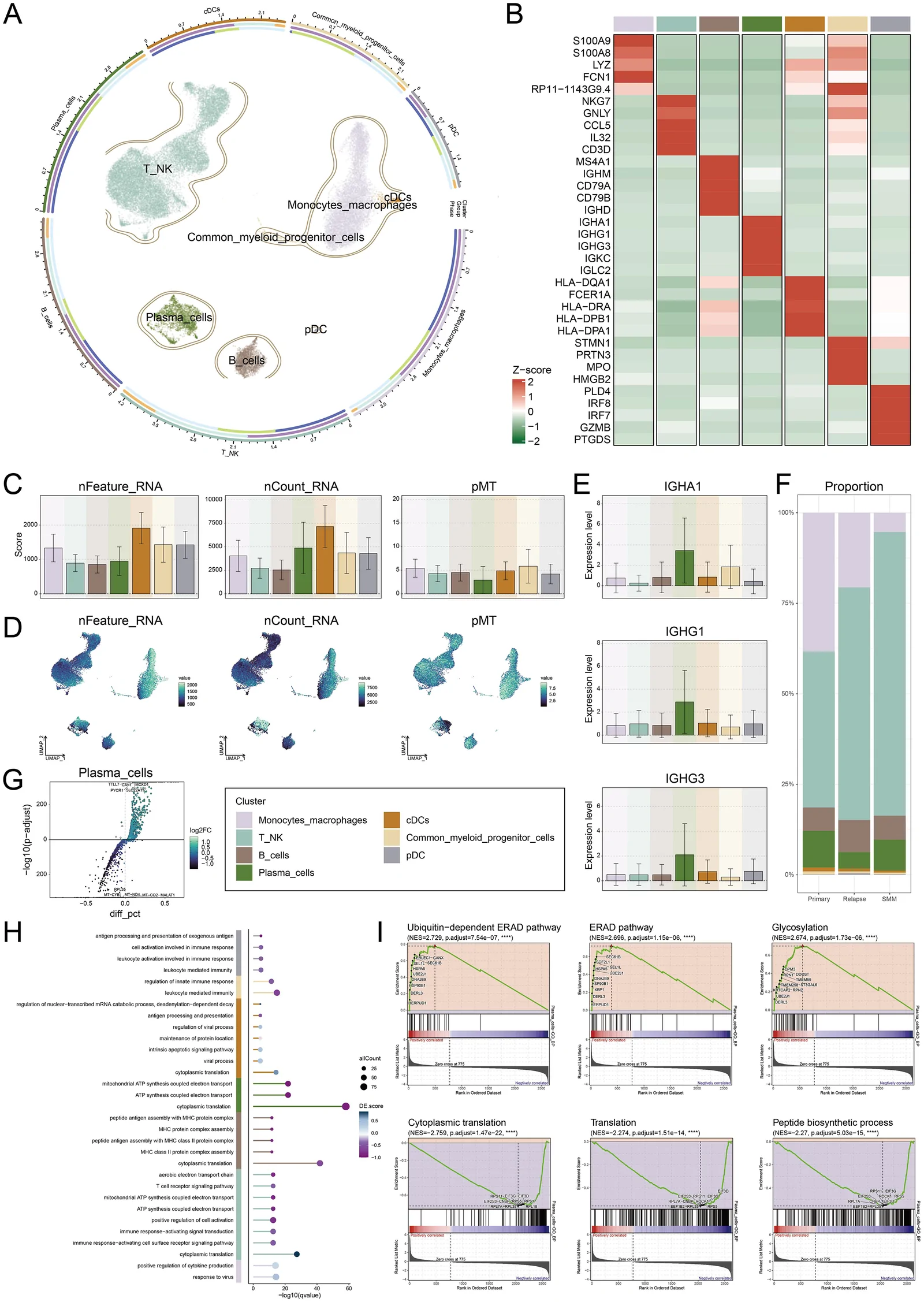
Heterogeneity of cells in SMM, NDMM, and RRMM. **(A)** UMAP plot showed 7 different cell types. **(B)** Heatmap showed the expression of top 5 DEGs across the 7 different cell types. **(C)** Bar plots showed the nFeature RNA, nCount RNA, and pMT scores for 7 different cell types. **(D)** UMAP plots displayed the values of nFeature RNA, nCount RNA and pMT scores. **(E)** Bar plots showed the expression of the top 3 genes of plasma cells, analyzed individually across all cell types. **(F)** The stacked bar graphs illustrated the relative amounts and distributions of 7 cell types among different disease subtypes. “Primary” refers to NDMM, while “Relapse” refers to RRMM. **(G)** Volcano plots demonstrated the expression of upregulated and downregulated DEGs in Plasma cells. **(H)** GO enrichment analysis of DEGs among 7 different cell types. **(I)** GSEA analyzed 3 positively enriched and 3 negatively enriched pathways in Plasma cells.

First, we presented the overall workflow of this study (**Figure 1**). Through the analysis of scRNA-seq data derived from 12 bone marrow samples of myeloma patients, we successfully distinguished 3 disease subtypes, namely SMM, NDMM, and RRMM cells. After strict quality control and batch effect elimination, 35,965 high-quality cells were isolated for subsequent analysis. Further dimensionality reduction and clustering analysis categorized these cells into 7 major cell populations: monocytes/macrophages, T/NK cells, B cells, plasma cells, conventional dendritic cells (cDCs), common myeloid progenitors (CMPs), and plasmacytoid dendritic cells (pDCs) (**Figure 2A**). Heatmaps were used to illustrate the expression profiles of canonical DEGs associated with each cell type (**Figure 2B**). Bar plots and UMAP plots were used to display nFeature RNA, nCount RNA, and percent mitochondrial transcripts (pMT) scores across the 7 cell populations. Notably, cDCs and plasma cells showed relatively high nCount RNA levels, indicating higher detected transcript counts in these cell types (**Figures 2C, D**). Bar plots illustrated the expression of the top 3 differential genes (*IGHA1*, *IGHG1*, and *IGHG3*) in plasma cells, which showed robust expression in this cell type (**Figure 2E**). Subsequently, we evaluated the distribution of cell type proportions across different disease subtypes (**Figure 2F**). Volcano plots revealed that the upregulated genes in plasma cells included *TTLL7*, *CAV1*, *MOXD1*, *PYCR1*, and *SLC22A17*, while the downregulated genes were *RPL35*, *MT-CYB*, *MT-ND4*, *MT-CO2*, and *MALAT1* (**Figure 2G**). Based on these DEGs, we conducted enrichment analysis of associated biological processes in distinct cell populations (**Figure 2H**). Plasma cells were enriched in biological processes such as cytoplasmic translation, ATP synthesis-coupled electron transport, and mitochondrial ATP synthesis-coupled electron transport. GSEA identified several top-ranked molecular pathways. The ubiquitin-dependent ERAD pathway, ERAD pathway, and glycosylation processes were positively enriched in plasma cells. In contrast, cytoplasmic translation, translation, and peptide biosynthetic processes were negatively enriched in this cell type (**Figure 2I**).

### 3.2 Single-cell characteristics of plasma cell subpopulations

**Figure 3:**
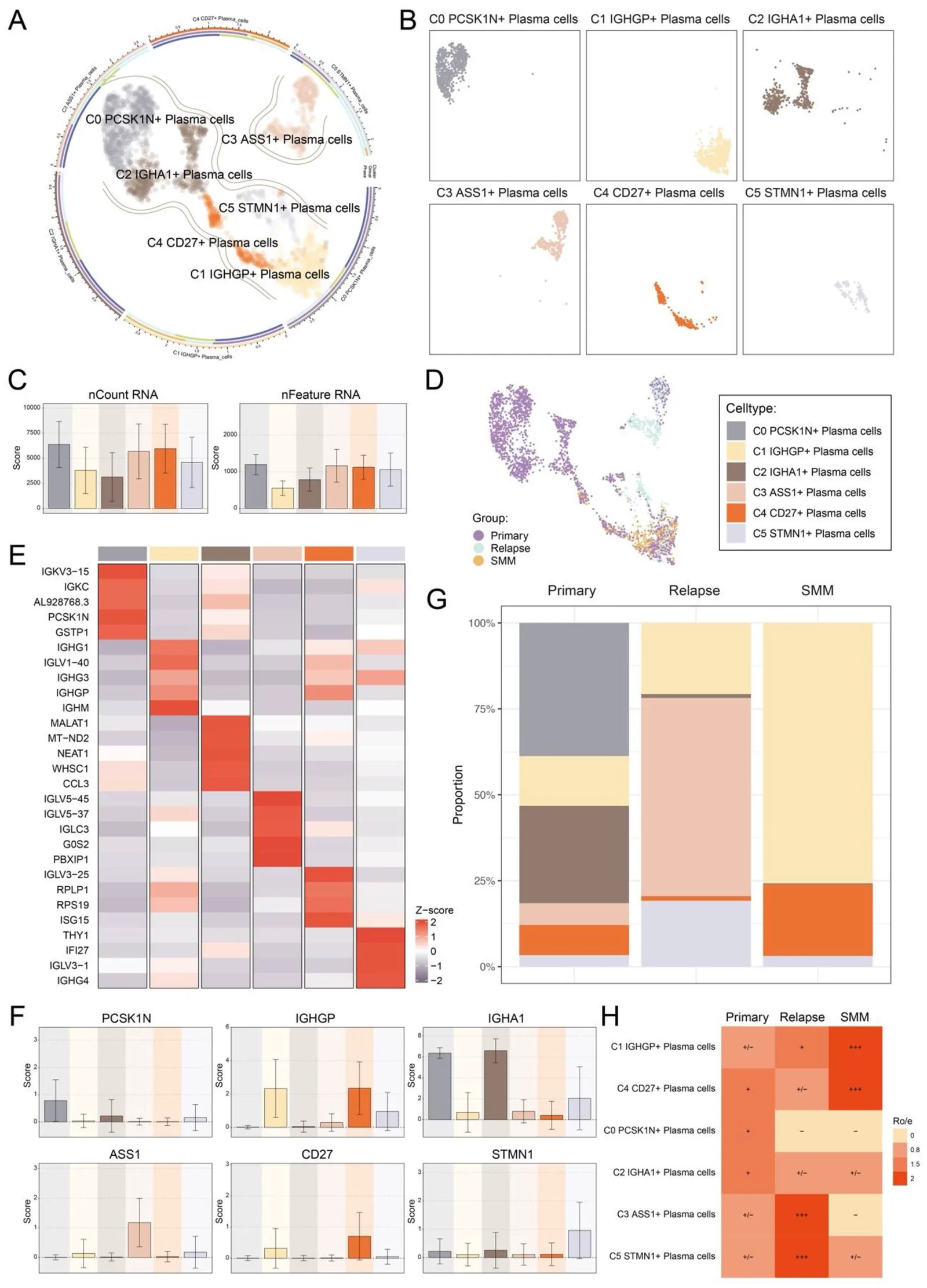
Heterogeneity of plasma cell subpopulations in SMM, NDMM, and RRMM. **(A)** UMAP plot showed 6 different plasma cell subpopulations. **(B)** Faceted UMAP plots illustrated the distribution characteristics of 6 plasma cell subpopulations, respectively. **(C)** Bar plots showed the nCount RNA and nFeature RNA scores of the 6 plasma cell subpopulations. **(D)** UMAP plot displayed the plasma cells colored according to disease subtypes. **(E)** Heatmap showed the expression of top 5 DEGs across the 6 plasma cell subpopulations. **(F)** Bar plots showed the expression of the named gene for each plasma cell subpopulation. **(G)** Stacked bar graphs illustrated the proportions of 6 plasma cell subpopulations. **(H)** Ro/e scores were used to assess the preferences of each cell type across different disease subtypes (SMM, NDMM, and RRMM). “Primary” refers to NDMM, while “Relapse” refers to RRMM.

To better characterize plasma cells in SMM, NDMM, and RRMM, we stratified plasma cells into 6 distinct subpopulations: C0 (*PCSK1N*+), C1 (*IGHGP*+), C2 (*IGHA1*+), C3 (*ASS1*+), C4 (*CD27*+), and C5 (*STMN1*+) plasma cells (**Figures 3A, B**). Subsequently, we assessed the nCount RNA and nFeature RNA values across all plasma cell subpopulations (**Figure 3C**). C2 *IGHA1*+ plasma cells exhibited low levels of both nCount RNA and nFeature RNA. Notably, C3 *ASS1*+ plasma cells displayed significantly higher nFeature RNA values. Analysis of plasma cell distribution across disease subtypes revealed that the majority of plasma cells were derived from NDMM samples, with relatively lower proportions in SMM and RRMM (**Figure 3D**). **Figure 3E** depicted the expression of the top 5 DEGs across the 6 plasma cell subpopulations. For C3 *ASS1*+ plasma cells, the top 5 DEGs were identified as *IGLV5-45*, *IGLV5-37*, *IGLC3*, *G0S2*, and *PBXIP1*. **Figure 3F** illustrated the expression patterns of named genes for each plasma cell subpopulation. Most plasma cell subpopulations showed high expression of their respective genes. ASS1 was highly expressed in C3 *ASS1*+ plasma cells, while IGHGP and CD27 were predominantly expressed in C1 *IGHGP*+ and C4 *CD27*+ plasma cells. IGHA1 was upregulated in both C0 *PCSK1N*+ and C2 *IGHA1*+ plasma cells, PCSK1N was enriched in C0 *PCSK1N*+ plasma cells, and STMN1 was highly expressed in C5 *STMN1*+ plasma cells. **Figure 3G** presented the proportional distribution of plasma cell subpopulations across individual patient samples. Notably, C1 *IGHGP*+ plasma cells were detected in all patient samples. With respect to disease subtype preference, C3 *ASS1*+ and C5 *STMN1*+ plasma cell subpopulations were more prevalent in RRMM, whereas C1 *IGHGP*+ and C4 *CD27*+ subpopulations were predominantly enriched in SMM (**Figure 3H**).

### 3.3 DEGs and enrichment analysis of plasma cell subpopulations

**Figure 4:**
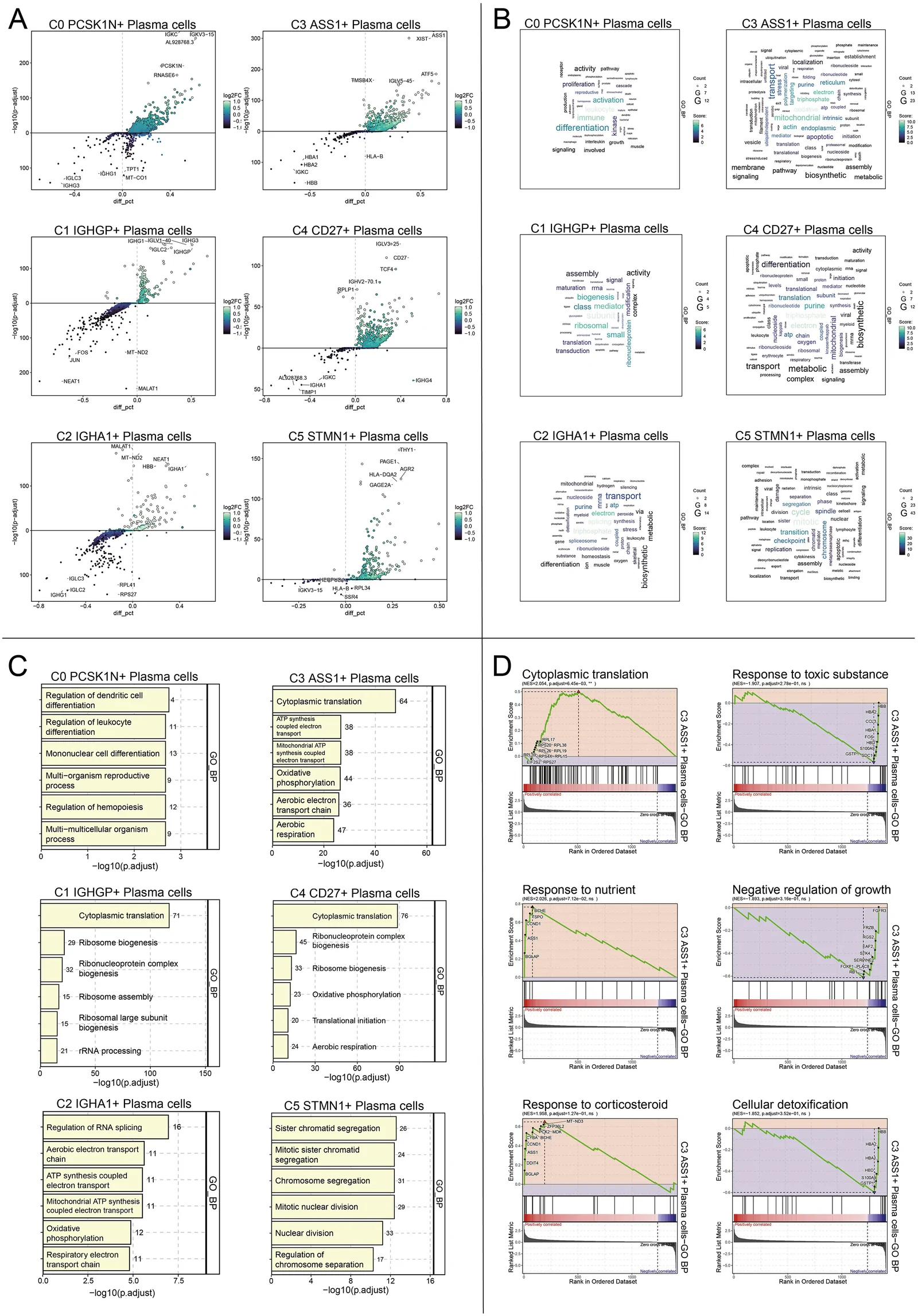
Enrichment analysis of plasma cell subpopulations. **(A)** Volcano plots showed the top 5 upregulated and top 5 downregulated DEGs in each plasma cell subpopulation. **(B)** Word cloud plots represented the functional enrichment results of the DEGs across plasma cell subpopulations. **(C)** Bar plots showed the GOBP enrichment results for DEGs in each plasma cell subpopulation. **(D)** GSEA results demonstrated the top 3 positively and top 3 negatively enriched pathways in the C3 *ASS1*+ plasma cell subpopulation.

To further explore the heterogeneity of plasma cells in SMM, NDMM, and RRMM, we analyzed the DEGs of the 6 plasma cell subpopulations and their functional enrichment profiles. The upregulated and downregulated genes in each plasma cell subpopulation were visualized via volcano plots (**Figure 4A**). Specifically, the predominant upregulated genes in C3 *ASS1*+ plasma cells included *ASS1*, *XIST*, *ATF5*, *IGLV5-45*, and *TMSB4X*, whereas the major downregulated genes were *IGKC*, *HBB*, *HBA2*, *HBA1*, and *HLA-B*. For C5 *STMN1*+ plasma cells, the key upregulated genes were *THY1*, *PAGE1*, *AGR2*, *HLA-DQA2*, and *GAGE2A*, while the main downregulated genes were *IGKV3-15*, *HERPUD1*, *HLA-B*, *SSR4*, and *RPL34*.

To elucidate the biological functions and molecular features of distinct plasma cell subpopulations, we performed Gene Ontology Biological Process (GOBP) enrichment analysis (**Figure 4B**). Word cloud plots revealed that C0 *PCSK1N*+ plasma cells were enriched in pathways associated with leukocyte, immune, activation and differentiation. C1 *IGHGP*+ plasma cells were enriched in pathways related to subunit, ribosomal and mediator, while C2 *IGHA1*+ plasma cells were enriched in pathways involving splicing, triphosphate, and electron. C3 *ASS1*+ plasma cells were enriched in pathways associated with oxidative, triphosphate, mitochondrial, electron, targeting, actin, purine, and intrinsic. C4 *CD27*+ plasma cells were enriched in pathways related to triphosphate, electron, purine, and ATP, and C5 *STMN1*+ plasma cells were enriched in pathways involving mitotic, cycle, transition, segregation, and checkpoint.

Subsequent cross-subpopulation enrichment analysis revealed that distinct subpopulations shared specific biological functions. For example, ribosome biogenesis and ribonucleoprotein complex biogenesis were commonly enriched in both C1 and C4 plasma cell subpopulations. Furthermore, pathways including cytoplasmic translation, ATP synthesis coupled electron transport, mitochondrial ATP synthesis coupled electron transport, oxidative phosphorylation, aerobic electron transport chain, and aerobic respiration were significantly enriched in C3 *ASS1*+ plasma cells (**Figure 4C**).

Furthermore, GSEA results (**Figure 4D**) demonstrated that the C3 *ASS1*+ plasma cell subpopulation was positively enriched in pathways related to cytoplasmic translation, response to nutrient, and response to corticosteroid. In contrast, this subpopulation was negatively enriched in pathways involving response to toxic substance, negative regulation of growth, and cellular detoxification.

**Figure 5:**
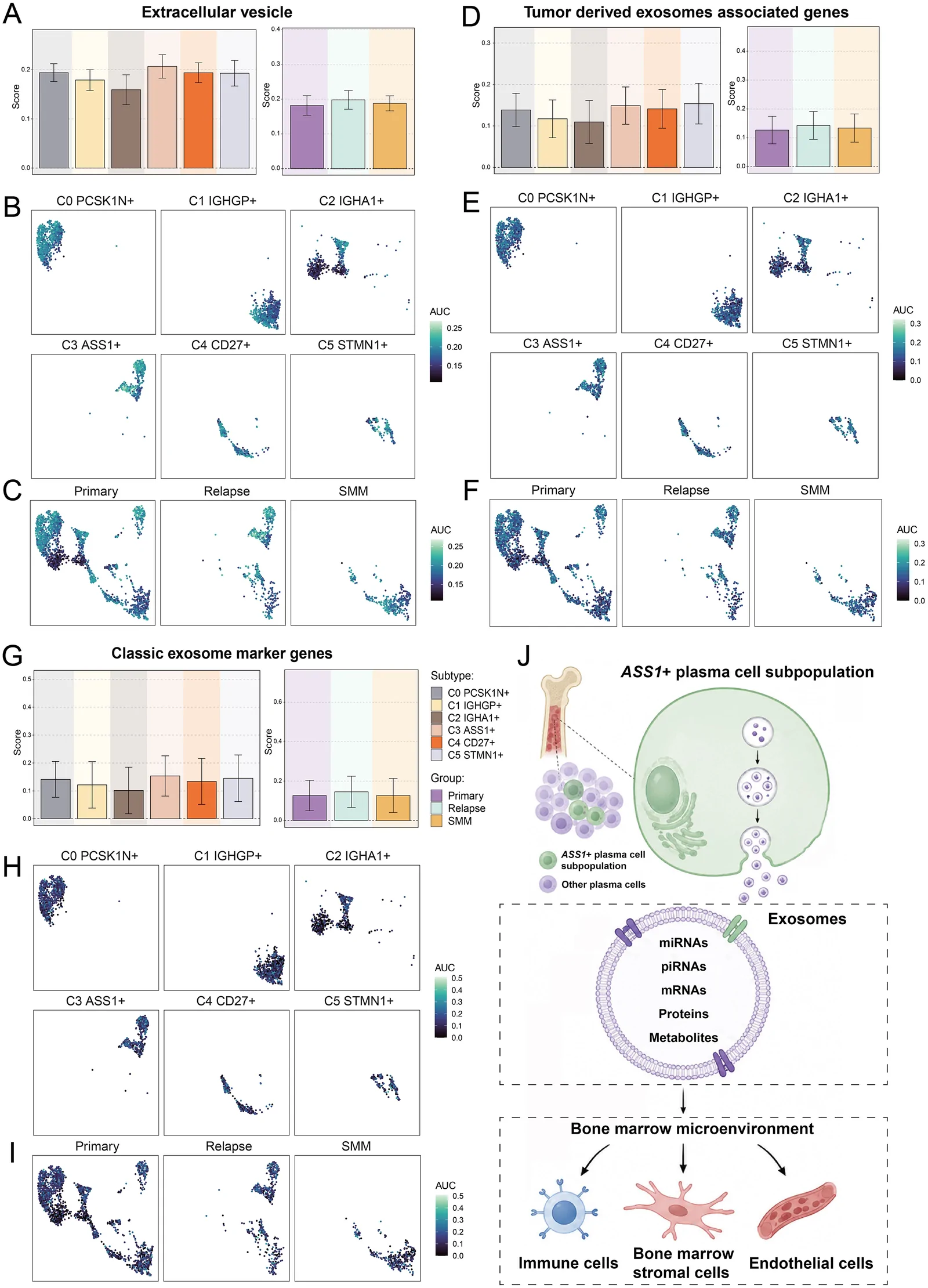
**(A)** Bar plots showed the EV scores across 6 plasma cell subpopulations (left) and 3 disease subtypes (right). **(B)** Faceted UMAP plots illustrated the distribution of EV scores across 6 plasma cell subpopulations, respectively. **(C)** Faceted UMAP plots illustrated the distribution of EV scores across 3 disease subtypes, respectively. **(D)** Bar plots showed the scores of tumor-derived exosomes-associated genes across 6 plasma cell subpopulations (left) and 3 disease subtypes (right). **(E)** Faceted UMAP plots illustrated the distribution of the scores of tumor-derived exosomes-associated genes across 6 plasma cell subpopulations, respectively. **(F)** Faceted UMAP plots illustrated the distribution of the scores of tumor-derived exosomes-associated genes across 3 disease subtypes, respectively. **(G)** Bar plots showed the scores of classic exosome marker genes across 6 plasma cell subpopulations (left) and 3 disease subtypes (right). **(H)** Faceted UMAP plots illustrated the distribution of the scores of classic exosome marker genes across 6 plasma cell subpopulations, respectively. **(I)** Faceted UMAP plots illustrated the distribution of the scores of classic exosome marker genes across 3 disease subtypes, respectively. **(J)** The hypothetical schematic model depicted regulations of exosomes secreted by C3 *ASS1*+ plasma subpopulation on the BMME.

Subsequently, we calculated the AUCell scores of multiple EV-related gene sets across 6 plasma cell subpopulations and 3 disease subtypes to characterize the EV-related genes of MM at the single-cell level. As shown in **Figure 5A** and **Figure 5B**, compared with other plasma cell subpopulations, the C3 *ASS1*+ plasma cell subpopulation had higher levels of EV genes collected from public databases. Meanwhile, RRMM presented higher levels of EV genes than SMM and NDMM (**Figures 5A, C**). Next, we adopted 2 additional gene sets to calculate exosome scores of plasma cells. Consistent with the above scoring results, the C3 *ASS1*+ plasma cell subpopulation had higher levels of tumor-derived exosomes-associated genes (**Figures 5D, E**) and classic exosome marker genes (**Figures 5G, H**). Likewise, RRMM also had higher levels of tumor-derived exosomes-associated genes (**Figures 5D, F**) and classic exosome marker genes (**Figures 5G, I**). These findings indicate that EV/exosome-related signatures are important features of the C3 *ASS1*+ plasma cell subpopulation and RRMM.

### 3.4 Pseudotime trajectory analyses of plasma cell subpopulations

**Figure 6:**
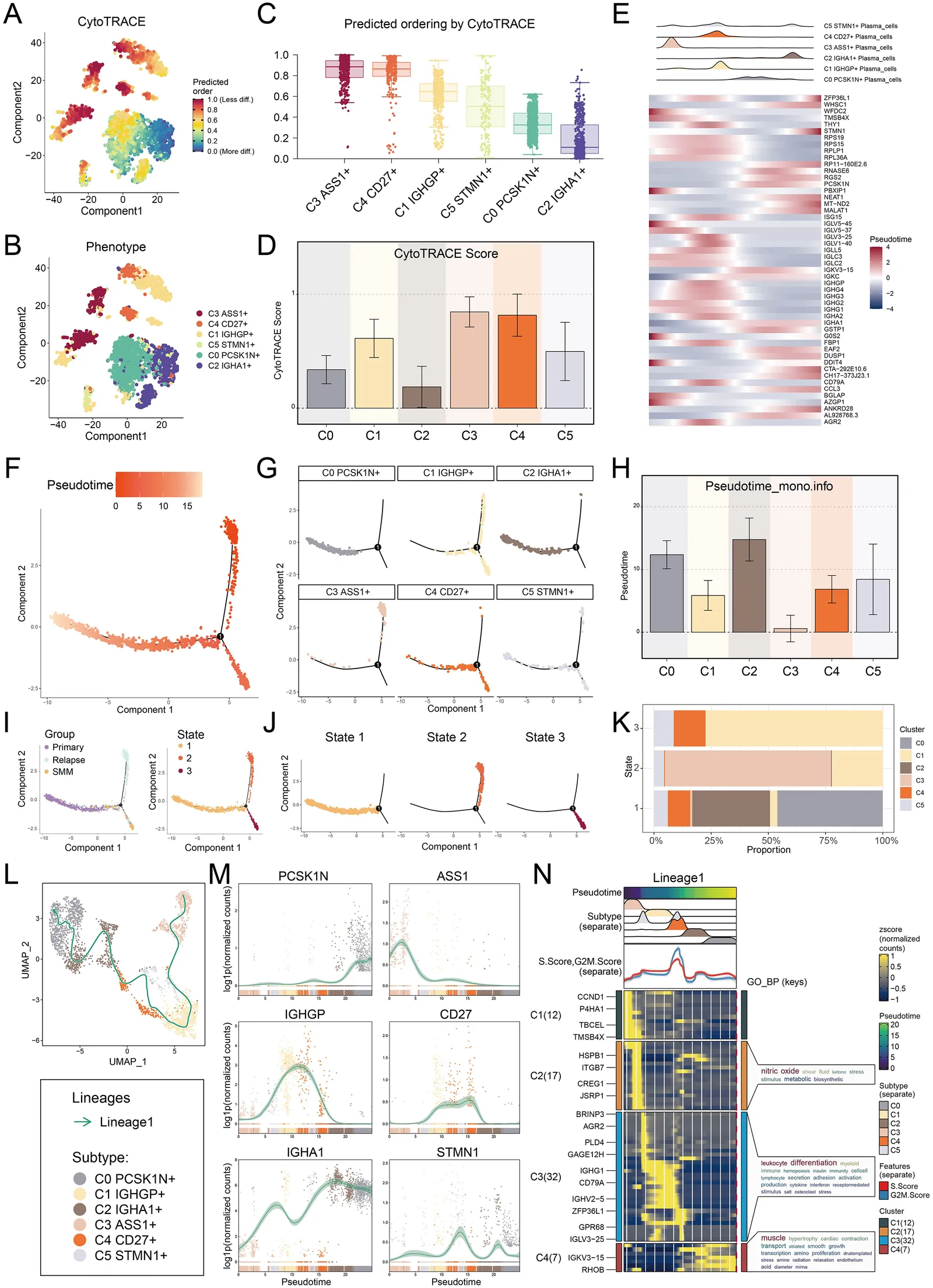
Pseudotime heterogeneity in plasma cell subpopulations. **(A, B)** UMAP plots depicted the stemness capacity of 6 plasma cell subpopulations assessed by CytoTRACE. **(C)** Plasma cell subpopulations were ranked using CytoTRACE, with higher scores indicating greater stemness and differentiation potential. **(D)** The bar plot illustrated the stemness capacity results of 6 plasma cell subpopulations assessed by CytoTRACE. **(E)** The Heatmap exhibited the temporal expression patterns of DEGs across plasma cell subpopulations along the Monocle-inferred pseudotime trajectory. **(F)** Pseudotime trajectory of integrated plasma cells inferred by Monocle, with cells colored according to pseudotime progression. **(G)** 2D trajectory faceted plots demonstrated the distribution of each plasma cell subpopulation across the pseudotime trajectories. **(H)** Bar plot showed differences in the predicted temporal order of 6 plasma cell subpopulations. **(I)** The same trajectory colored by the disease stage (left) and transcriptional state (right), showing 3 distinct pseudotime-defined cell states. “Primary” refers to NDMM, while “Relapse” refers to RRMM. **(J)** 2D trajectory faceted plots depicted the distribution of plasma cells colored by pseudotime-defined cell states. **(K)** Stacked bar plots illustrated the proportion of 6 plasma cell subpopulations in the 3 pseudotime-defined cell states. **(L)** The UMAP plot demonstrated the temporal dynamics of cell differentiation profiles of 6 plasma cell subpopulations. **(M)** The distribution of named genes for different plasma cell subpopulations along Slingshot-simulated pseudotime trajectories. **(N)** The heatmap depicted GOBP pathway enrichment during plasma cell differentiation.

To infer the differentiation process and developmental trajectory of plasma cell subpopulations, clarify their interrelationships during differentiation and development, and assess their differentiation capacity, we applied a series of trajectory prediction strategies. Initially, CytoTRACE was used to evaluate the differentiation potential of plasma cells. Our results indicated that C3 *ASS1*+ plasma cells showed the highest CytoTRACE scores, indicating a stronger stemness-like transcriptional state. The remaining subpopulations exhibited differentiation potential in the descending order of C4 (*CD27*+), C1 (*IGHGP*+), C5 (*STMN1*+), C0 (*PCSK1N*+), and C2 (*IGHA1*+) plasma cells (**Figures 6A–D**).

Subsequently, we performed pseudotime trajectory analysis on the integrated plasma cells from all disease stages using Monocle to capture the dynamic transcriptional alterations associated with plasma cell evolution (**Figure 6F**). C3 *ASS1*+ plasma cells were clearly observed to be predominantly distributed in the early stages of differentiation (**Figures 6G, H**). **Figure 6E** depicted the temporal variations of DEGs along the predicted pseudotime trajectory. Overlaying disease stages onto the trajectory identified a directional transition, with the initial branches populated primarily by plasma cells from RRMM (**Figure 6I**). Furthermore, this analysis revealed a branch point in the predicted developmental trajectory of plasma cells, which split the entire trajectory into 3 distinct states (**Figure 6J**). Throughout the pseudotime trajectory, C3 *ASS1*+ plasma cells were mainly localized in state 2 (**Figure 6K**).

Finally, we repeated the developmental trajectory analysis using Slingshot (**Figure 6L**) and inferred a putative transcriptional trajectory: C3 (*ASS1*+) plasma cells → C5 (*STMN1*+) plasma cells → C1 (*IGHGP*+) plasma cells → C4 (*CD27*+) plasma cells → C2 (*IGHA1*+) plasma cells → C0 (*PCSK1N*+) plasma cells (putative pseudotime ordering). This finding further corroborated the conclusions derived from the Monocle analysis. Along the pseudotime axis of plasma cells, IGHGP and CD27 were predominantly expressed in the middle stage of the developmental trajectory, while STMN1 was mainly expressed in the mid-late stage. In contrast, ASS1 exhibited high initial expression that gradually declined with pseudotime, whereas PCSK1N and IGHA1 showed a steady increase in expression from the early to the late stages (**Figure 6M**). Based on the characteristics of cell lineage development, GOBP enrichment analysis was further performed (**Figure 6N**).

In summary, our findings indicate that C3 *ASS1*+ plasma cells are poorly differentiated malignant tumor cells, which play a crucial role in the progression of MM.

### 3.5 Analysis of cell interaction communication patterns in MM

**Figure 7:**
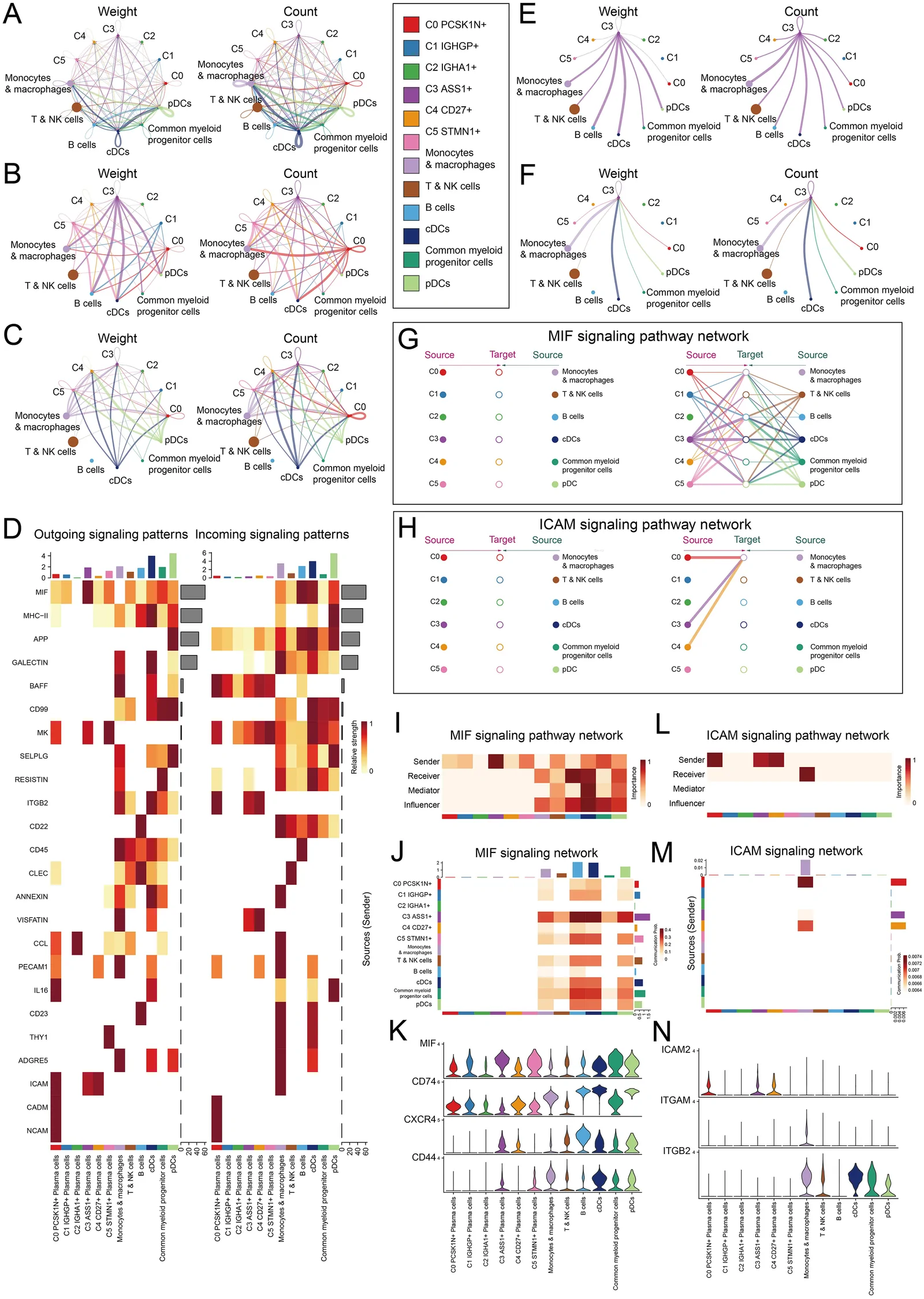
Intercellular communication profiles of plasma cell subpopulations. **(A)** Circle plots of intercellular communication networks for all cell types. Left: network based on interaction weights, edge thickness indicating weights; right: network on interaction counts, edge thickness matching event numbers. Circle size correlated with group cell counts. **(B)** Circle plots showed interaction intensity (left) and count (right) of plasma cell subpopulations as signal sources. **(C)** Circle plots presented interaction intensity (left) and count (right) of plasma cell subpopulations as signal targets. **(D)** Dual heatmaps visualized cellular signaling. Left: outgoing signals of each cell type (color depth positively correlating with activity); right: incoming signals, reflecting cell signal-receiving capacity. **(E)** Circle plots demonstrated interaction intensity (left) and count (right) of C3 plasma cell subpopulation as signal source. **(F)** Circle plots depicted interaction intensity (left) and count (right) of C3 plasma cell subpopulation as signal target. **(G, H)** Hierarchical plots of MIF and ICAM pathways revealed autocrine/paracrine interactions between 6 plasma cell subpopulations and other cells. Filled circles = source cells; open circles = target cells. **(I, L)** Centrality network heatmaps of MIF and ICAM pathways showed regulatory importance of functional roles. Color gradients indicated centrality levels. **(J, M)** Heatmap demonstrated the communication probability of different cell clusters in the MIF and ICAM signaling communication network. **(K, N)** Violin plots depicted intercellular interactive proteins in MIF and ICAM signaling pathways.

To infer and characterize the intercellular communication between plasma cell subpopulations and other cell types in MM, we employed CellChat on single-cell sequencing data. Circle diagrams comprehensively summarized the intensity and quantity of interactions across all cell types (**Figure 7A**). Subsequently, circle graphs quantified the interaction intensity and count between all cells when plasma cells served as the signal source (**Figure 7B**) and target (**Figure 7C**), respectively. Notably, a robust intercellular communication network was identified between C3 *ASS1*+ plasma cells and monocytes/macrophages, cDCs, as well as pDCs.

Following the identification of signaling pathways underlying each cellular interaction, we focused on the signaling crosstalk between C3 *ASS1*+ plasma cells and other cell types. Initially, we analyzed the interaction intensity and count between C3 *ASS1*+ plasma cells and other cell subpopulations (**Figures 7E, F**). These results also demonstrated a strong intercellular communication network between C3 *ASS1*+ plasma cells, monocytes/macrophages, and cDCs.

To further characterize the key incoming and outgoing signals between cells, we evaluated the contributions of incoming and outgoing signaling patterns using heatmaps. As shown in **Figure 7D**, the MIF and ICAM signaling pathways contributed substantially to the communication of C3 *ASS1*+ plasma cells, primarily through interactions with other cell types.

Subsequently, we focused on the MIF signaling network. Our results showed that the C0, C1, C3, C4, and C5 plasma cell subpopulations exhibited high activity, with C3 *ASS1*+ plasma cells being the most prominent (**Figure 7G**). Notably, plasma cells were capable of engaging in paracrine interactions with other cell types. Network centrality scoring revealed that C3 *ASS1*+ plasma cells were inferred as major signal senders in the CellChat model, while cDCs primarily acted as signal receivers, mediators, and influencers (**Figure 7I**). A heatmap depicted the interaction probabilities between different cell types in response to MIF (**Figure 7J**). Additionally, violin plots indicated that MIF and CD74 transcripts showed higher expression patterns contributing to inferred MIF signaling (**Figure 7K**).

We then investigated the ICAM signaling network. The results demonstrated that the C0, C3, and C4 plasma cell subpopulations could engage in paracrine interactions with monocytes/macrophages (**Figure 7H**). Network centrality scoring indicated that C0, C3, and C4 plasma cells acted as signal senders, whereas monocytes/macrophages served as signal receivers (**Figure 7L**). A heatmap illustrated the interaction probabilities between different cell types in response to ICAM (**Figure 7M**). Moreover, violin plots showed high activity of ICAM2 and ITGB2 proteins in the ICAM signaling pathway (**Figure 7N**).

### 3.6 Analysis of TFs regulatory modules and metabolic pathways

**Figure 8:**
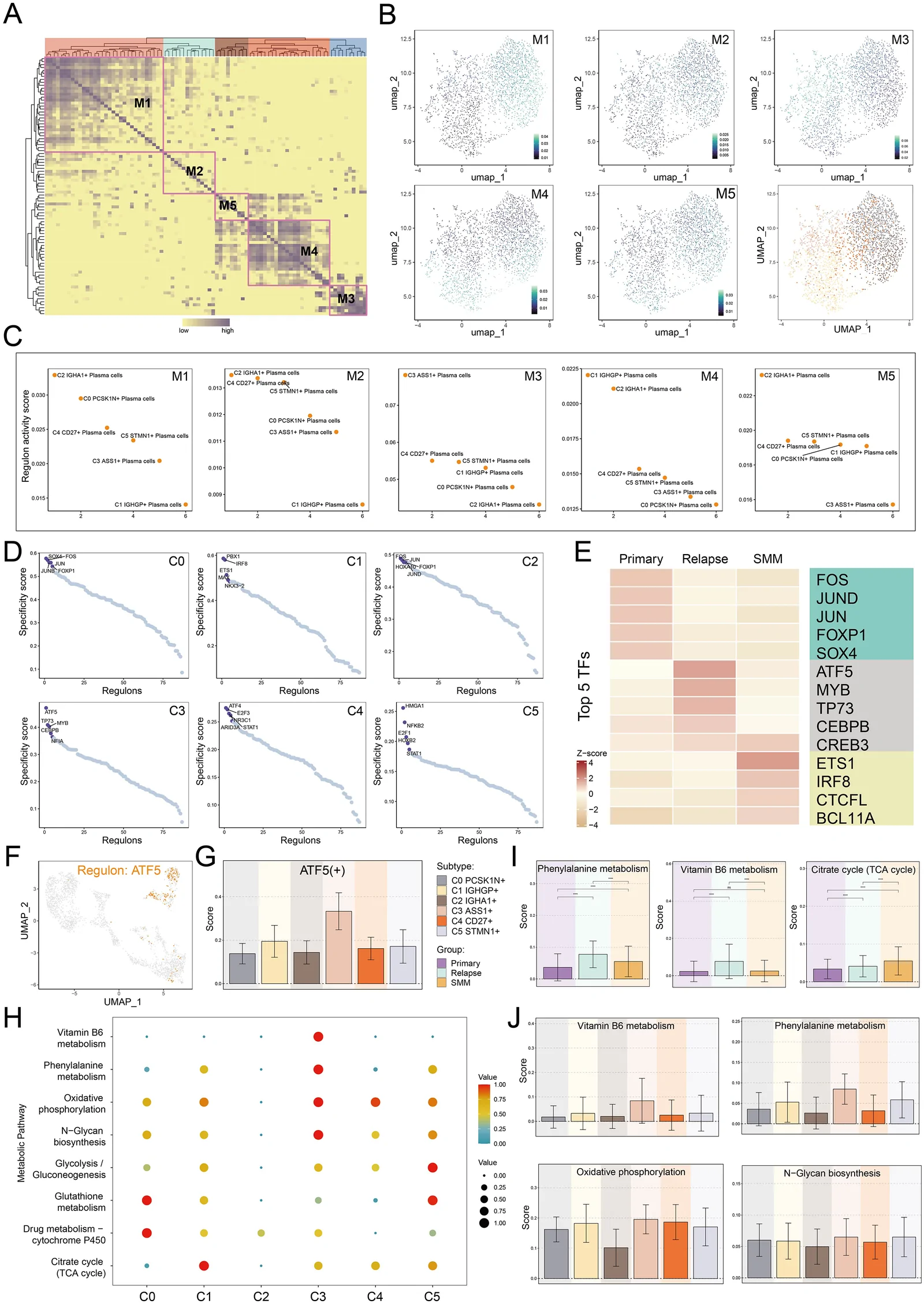
TFs regulatory network and metabolism-related pathways in plasma cell subpopulations. **(A)** The heatmap illustrated the similarity of regulatory modules based on SCENIC-identified modules and AUCell scores, showing five regulatory modules (M1, M2, M3, M4, and M5) across all plasma cell subpopulations. **(B)** UMAP plots depicted the distribution of the M1, M2, M3, M4, and M5 modules across 6 plasma cell subpopulations. UMAP plots colored and visualized all plasma cells based on the activity scores of regulatory modules according to 6 plasma cell subpopulations (the bottom-right panel). **(C)** The ranking of 6 plasma cell subpopulations in each module (M1-M5) was depicted based on the Regulon Activity Score (RAS). **(D)** The ranking of TFs in each plasma cell subpopulation (C0-C5) was illustrated based on the Regulatory Specificity Score (RSS). **(E)** The heatmap displayed the differential expression of the top 5 TFs across 3 disease stages (SMM, NDMM, and RRMM). **(F)** The UMAP plot showed the distribution of the ATF5 regulon across all plasma cells. **(G)** The bar plot illustrated the score of ATF5 regulon across 6 plasma cell subpopulations. **(H)** The bubble plot displayed the scores of top metabolism-related pathways across 6 plasma cell subpopulations. **(I)** Bar plots illustrated the AUCell values of 3 metabolism-related pathways across different disease stages (SMM, NDMM, and RRMM). “Primary” refers to NDMM, while “Relapse” refers to RRMM. **(J)** Bar plots depicted the AUCell values of the top metabolism-related pathways across 6 plasma cell subpopulations.

TFs regulate gene transcription by binding to specific nucleotide sequences upstream of target genes, thereby modulating cellular biological functions. Based on the heatmap of TFs correlation (**Figure 8A**), we clustered TFs with similar functional characteristics and expression patterns into 5 major modules, designated as M1, M2, M3, M4, and M5. Subsequently, we visualized the clustered modules and assessed the expression levels of each plasma cell subpopulation across different modules using UMAP plots (**Figure 8B**). The results demonstrated that C3 *ASS1*+ plasma cells exhibited significant expression in the M3 module. To further validate this observation, we performed transcription factor regulatory activity score analysis. We observed that within the M3 module, C3 *ASS1*+ plasma cells displayed the highest transcription factor regulatory activity score, followed by C4, C5, C1, C0, and C2 plasma cell subpopulations (**Figure 8C**). Furthermore, we analyzed the top 5 TFs in each plasma cell subpopulation. Specificity scoring identified ATF5, TP73, MYB, CEBPB, and NFIA as the top 5 TFs in C3 *ASS1*+ plasma cells (**Figure 8D**). **Figure 8E** showed that the top 5 TFs in RRMM were ATF5, MYB, TP73, CEBPB, and CREB3, while those in SMM were ETS1, IRF8, CREB3, CTCFL, and BCL11A. For NDMM, the top 5 TFs were FOS, JUND, JUN, FOXP1, and SOX4. Finally, we visualized the distribution characteristics of the ATF5 regulon using UMAP plots (**Figure 8F**), which showed the highest expression in C3 *ASS1*+ plasma cells, followed by C1, C5, C4, C2, and C0 plasma cell subpopulations (**Figure 8G**).

To further explore the metabolic characteristics of plasma cell subpopulations, we examined metabolic pathways and identified key processes, including vitamin B6 metabolism, phenylalanine metabolism, oxidative phosphorylation, N-glycan biosynthesis, glycolysis/gluconeogenesis, glutathione metabolism, drug metabolism-cytochrome P450, and the citrate cycle (TCA cycle) (**Figure 8H**). Phenylalanine metabolism and vitamin B6 metabolism exhibited higher activity in RRMM, while the citrate cycle (TCA cycle) was upregulated in SMM (**Figure 8I**). Notably, vitamin B6 metabolism, phenylalanine metabolism, oxidative phosphorylation, and N-glycan biosynthesis displayed higher activity in C3 *ASS1*+ plasma cells (**Figure 8J**).

### 3.7 ASS1 sustains malignant phenotypes in MM

Guided by our single-cell transcriptomic analysis, which highlighted *ASS1* as a malignancy-associated gene enriched in tumor cell subpopulations with proliferative and aggressive features, we next performed loss-of-function experiments to determine whether ASS1 is functionally required to sustain cancer cell fitness. Because ASS1 is a key metabolic enzyme implicated in arginine/citrulline flux and has been linked to tumor growth advantages across malignancies, we selected two widely used human MM cell lines, RPMI 8226 and U266, as tractable *in vitro* systems for loss-of-function experiments and quantitative functional readouts. Cells were transfected with two independent siRNAs targeting *ASS1* (si-ASS1#1 and si-ASS1#2) or a non-targeting control (si-Ctrl) to minimize off-target concerns and enable robust phenotype attribution.

*ASS1* silencing efficiency was first confirmed by qRT-PCR (**Supplementary Figure 1A**). In both RPMI 8226 and U266 cells, si-ASS1#1 and si-ASS1#2 markedly reduced *ASS1* mRNA levels compared with si-Ctrl, validating effective knockdown and providing the technical basis for subsequent functional assays.

**Figure 9:**
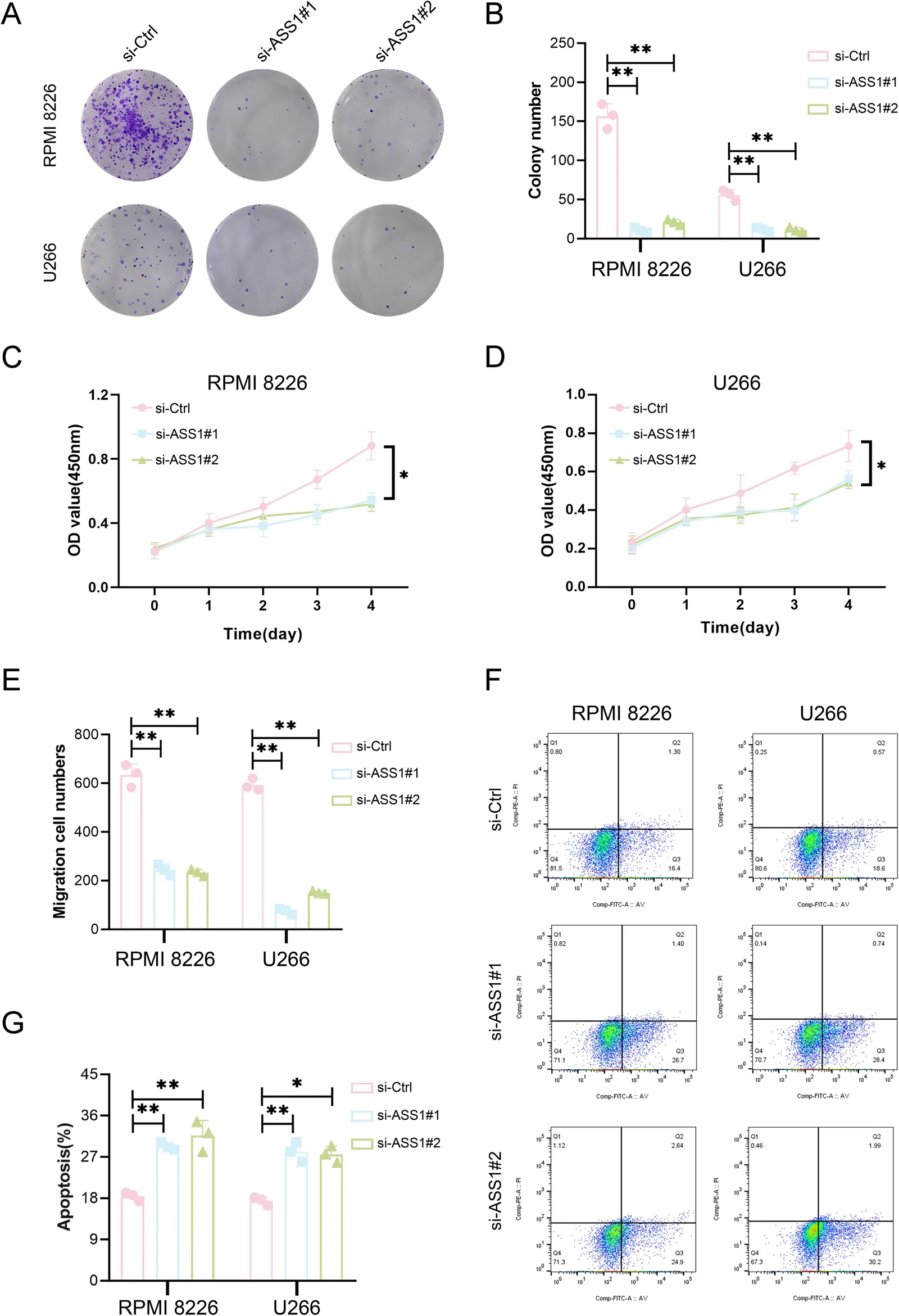
ASS1 knockdown suppresses proliferation and migration while promoting apoptosis in RPMI 8226 and U266 cells. **(A)** Representative images of colony formation assays in RPMI 8226 and U266 cells transfected with control siRNA (si-Ctrl) or two independent ASS1-targeting siRNAs (si-ASS1#1 and si-ASS1#2). **(B)** Quantification of colony numbers showed significantly reduced clonogenic capacity upon ASS1 silencing. **(C, D)** CCK-8 assays measured cell viability at indicated time points in RPMI 8226 **(C)** and U266 **(D)** cells. ASS1 knockdown markedly attenuated time-dependent growth compared with si-Ctrl. **(E)** Quantification of migrated cells demonstrated significantly decreased migratory capacity in si-ASS1 groups. **(F)** Representative Annexin V/PI flow cytometry plots showed apoptosis distribution in RPMI 8226 and U266 cells after ASS1 knockdown. **(G)** Statistical analysis of apoptotic rates indicated increased apoptosis upon ASS1 depletion. Data are presented as mean ± SD from at least 3 independent experiments. *P < 0.05, **P < 0.01 versus si-Ctrl.

We then evaluated long-term proliferative potential using colony formation assays, which reflect the capacity of single cells to survive and undergo sustained clonal expansion. Representative images showed a pronounced reduction in colony formation following *ASS1* depletion in both cell lines (**Figure 9A**), and quantitative analysis confirmed a significant decrease in colony numbers in si-ASS1 groups relative to si-Ctrl (**Figure 9B**). These data indicate that *ASS1* supports clonogenic survival, consistent with the single-cell inference that *ASS1*+ plasma cell subpopulations may possess enhanced growth competence.

To assess short-term growth kinetics, we performed CCK-8 assays. *ASS1* knockdown significantly blunted the time-dependent increase in OD450 in both RPMI 8226 and U266 cells (**Figures 9C, D**), indicating impaired proliferative expansion upon *ASS1* loss. Together with the clonogenic findings, these results support a growth-promoting role of ASS1 across both long-term and short-term proliferation metrics.

Because our single-cell analyses further suggested that ASS1 expression co-varies with tumor aggressiveness-related programs, we next examined cell motility using transwell migration assays. Compared with control cells, both si-ASS1 constructs substantially reduced the number of migrated cells in RPMI 8226 and U266 (**Figure 9E**). These data indicate that ASS1 contributes to migratory capacity in vitro.

Finally, to determine whether reduced growth was accompanied by altered cell survival, apoptosis was quantified by Annexin V/PI flow cytometry. Representative dot plots demonstrated an increased apoptotic fraction following *ASS1* knockdown (**Figure 9F**), and pooled analysis confirmed significantly higher apoptosis rates in si-ASS1 groups compared with si-Ctrl in both cell lines (**Figure 9G**). This indicates that ASS1 contributes to survival maintenance, and its depletion shifts cells toward programmed cell death.

Collectively, these functional data demonstrate that ASS1 is required to maintain proliferation, clonogenic capacity, and migration while restraining apoptosis in RPMI 8226 and U266 cells. Importantly, when integrated with the single-cell transcriptomic evidence implicating ASS1 in malignant, high-activity cellular subpopulations, our results support a model in which ASS1 is not merely a correlative marker but a functional determinant of tumor cell fitness, potentially through metabolic support of growth and survival programs.

## 4 Discussion

Through scRNA-seq analysis, we identified 7 major cell populations in the bone marrow of MM patients, including monocytes/macrophages, T/NK cells, B cells, plasma cells, cDCs, CMPs, and pDCs. Among these, plasma cells exhibited significant heterogeneity and were stratified into 6 distinct subpopulations (C0-C5) based on their gene expression profiles. Notably, C3 *ASS1*+ plasma cells displayed unique characteristics, including higher nFeature RNA levels, and were predominantly enriched in RRMM samples. ASS1 (argininosuccinate synthase 1) is a key enzyme involved in the urea cycle and arginine biosynthesis (59), and its overexpression has been associated with tumor progression and drug resistance in various cancers, such as ovarian cancer (60), NSCLC (61), and T-cell acute lymphoblastic leukemia (T-ALL) (62). Our results suggest that C3 *ASS1*+ plasma cells may represent a highly malignant subpopulation that contributes to MM progression and relapse. Interestingly, proteasome inhibitor treatment, which represents the therapeutic backbone of most RRMM regimens, paradoxically triggers the release of EVs enriched in cell cycle proteins and heparanase (63). These exosomes subsequently confer resistance on neighboring MM cells through pERK activation and extracellular matrix remodeling (63). It has also been reported that exposure to melphalan or bortezomib induces MM cells to secrete EVs containing elevated levels of acid sphingomyelinase (64). Such EVs have been demonstrated to counteract the cytotoxic effects of these drugs in treatment-naïve myeloma cells (64). Given that C3 *ASS1*+ plasma cells are predominantly enriched in RRMM patients who have likely received prior proteasome inhibitor therapy, we speculate that this plasma cell subpopulation may represent the dominant source population of EVs within the refractory BMME. Through AUCell scoring analysis based on multiple public gene sets, we consistently demonstrated that the C3 *ASS1*+ plasma cell subpopulation exhibited significantly higher enrichment of EV-related genes, tumor-derived exosome-associated genes, and canonical exosome marker genes compared with other plasma cell subpopulations. Moreover, disease subtype comparison further revealed that RRMM displayed markedly elevated EV signature levels relative to both SMM and NDMM. These single-cell transcriptomic findings solidly identify exosome biogenesis and secretion as a prominent molecular hallmark of the C3 *ASS1*+ plasma cell subpopulation and RRMM, providing novel insights into the cellular and molecular heterogeneity underlying MM progression. The hyperactive exosome signature of C3 *ASS1*+ plasma cells may endow this malignant subpopulation with the capacity to remodel the BMME through exosome secretion, thereby promoting MM progression (**Figure 5J**). Future studies should clarify whether *ASS1* directly regulates genes involved in EV biogenesis. Besides, the negative enrichment of response to toxic substance, negative regulation of growth, and cellular detoxification may enable C3 *ASS1*+ plasma cells to evade growth inhibition, reduce sensitivity to chemotherapeutic toxins, and enhance stress tolerance. Additionally, C5 *STMN1*+ plasma cells were also more prevalent in RRMM, and STMN1 (stathmin 1) is a well-known oncoprotein that regulates microtubule dynamics (65) and promotes cell proliferation and metastasis (66–70), further supporting the role of these subpopulations in disease aggressiveness.

In contrast, C1 *IGHGP*+ and C4 *CD27*+ plasma cells were predominantly enriched in SMM, suggesting that these subpopulations may be associated with the early stages of MM. CD27 is a key marker of B cell activation and plasma cell differentiation (71–73), and its expression has been linked to favorable prognosis in MM (74). The distinct distribution of plasma cell subpopulations across MM subtypes highlights the dynamic changes in cellular composition during disease progression, which may have important implications for clinical outcome and treatment response.

CytoTRACE and pseudotime trajectory analyses revealed that C3 *ASS1*+ plasma cells possessed the highest differentiation potential, indicating that they are low-differentiated tumor cells. The developmental trajectory inferred by Monocle and Slingshot demonstrated a linear lineage pathway: C3 (*ASS1*+) → C5 (*STMN1*+) → C1 (*IGHGP*+) → C4 (*CD27*+) → C2 (*IGHA1*+) → C0 (*PCSK1N*+). This trajectory suggests that C3 (*ASS1*+) plasma cells may serve as the root of the malignant clone. The gradual decline in ASS1 expression and increase in PCSK1N and IGHA1 expression along the pseudotime axis further supports this developmental pathway. PCSK1N (proprotein convertase subtilisin/kexin type 1 inhibitor) is involved in the regulation of protein processing, while IGHA1 is a major immunoglobulin isotype produced by plasma cells. The enrichment of C3 *ASS1*+ plasma cells in the early stages of the trajectory and their association with RRMM suggest that targeting this subpopulation may prevent disease progression and relapse.

CellChat analysis revealed a robust intercellular communication network between C3 *ASS1*+ plasma cells and other cell types in the BMME, particularly monocytes/macrophages and cDCs. The MIF and ICAM signaling pathways were identified as key mediators of this crosstalk. MIF (macrophage migration inhibitory factor) is a pro-inflammatory cytokine that plays a crucial role in tumor progression, angiogenesis, and immune suppression (75). Compared with MM patients maintaining sustained response, MIF expression was markedly increased in RRMM patients, and high MIF expression was significantly correlated with shorter progression-free survival (PFS) and overall survival (OS) (76). Our results showed that C3 *ASS1*+ plasma cells were the most active senders of MIF signals, while cDCs acted as key receivers and mediators. This suggests that C3 *ASS1*+ plasma cells may modulate the immune microenvironment through MIF signaling to promote immune suppression and tumor survival. Additionally, the ICAM signaling pathway, which is involved in cell adhesion and migration (77), was found to mediate communication between C0, C3, C4 plasma cells and monocytes/macrophages. ICAM2 and ITGB2, key proteins in the ICAM pathway, were highly active, indicating that cell adhesion may facilitate the interaction between plasma cells and the BMME, supporting tumor cell survival and proliferation. The crosstalk between MM cells and the BM niche involves not only direct cell–cell contact, but also a critical role played by MM-derived EVs. Targeting these cell-cell communication pathways may disrupt the supportive microenvironment and improve therapeutic outcomes (78–80).

SCENIC analysis identified 5 major TF regulatory modules, with C3 *ASS1*+ plasma cells exhibiting significant expression in the M3 module. The top 5 TFs in C3 *ASS1*+ plasma cells were ATF5, TP73, MYB, CEBPB, and NFIA. ATF5 (activating transcription factor 5) has been shown to promote cell survival and drug resistance in various cancers (81–83), while TP73 (tumor protein 73) is a member of the p53 family and plays a role in cell cycle regulation and apoptosis (84, 85). MYB (myeloblastosis oncogene) is a key regulator of hematopoietic cell proliferation and differentiation (86), and its dysregulation is associated with the progression in leukemia (87, 88). MYB and its interacting partners represent promising therapeutic targets in leukemia, and a number of MYB-targeted therapeutic agents have been designed and evaluated as potential anticancer candidates in preclinical research (89). Given that CEBPB can activate the super−enhancer of PPP1R15B to drive MM progression and that inhibition of PPP1R15B exerts anti-myeloma effects in combination with bortezomib (90), the CEBPB-PPP1R15B axis may serve as a key regulatory and therapeutic target in C3 *ASS1*+ plasma cells. Furthermore, CEBPB can also serve as a core regulator of noncoding RNA (ncRNA) networks, which exert essential functions in tumorigenesis, metastasis, and therapeutic resistance. Notably, microRNAs such as miR-21 and miR-146a, can be transferred via MM-derived EVs to bone marrow stromal cells (BMSCs), thereby promoting the secretion of interleukin-6 (IL-6) (91, 92). Besides, piwi-interacting RNA piRNA-823 is highly enriched in EVs derived from MM cells, especially in advanced-stage disease, promoting endothelial cell proliferation and invasion by upregulating VEGF, IL-6, and ICAM-1 expression (93). Future studies should examine whether EVs from C3 *ASS1*+ plasma cells carry functionally active piRNAs. These TFs may cooperatively regulate the gene expression program of C3 *ASS1*+ plasma cells, contributing to their malignant phenotype.

Metabolic pathway analysis revealed that vitamin B6 metabolism, phenylalanine metabolism, oxidative phosphorylation, and N-glycan biosynthesis were more active in C3 *ASS1*+ plasma cells. Vitamin B6 metabolism is involved in amino acid metabolism and energy production, and its dysregulation has been linked to tumor progression (94). Phenylalanine metabolism plays a role in protein synthesis and oxidative stress response, and its upregulation may contribute to MM cell survival (95). Oxidative phosphorylation is a key metabolic pathway for energy production, and enhanced oxidative phosphorylation has been associated with drug resistance in MM (96, 97). Recent studies have shown that tumor cells with high oxidative phosphorylation levels or under metabolic stress secrete EVs (98). These EVs can transfer mitochondrial components and metabolites to recipient cells, helping these cells develop drug resistance and improve their survival. We hypothesize that the C3 *ASS1*+ plasma cell subpopulation can secrete EVs with a unique molecular signature, due to its distinct metabolic and transcriptional profiles. These EVs then help MM develop resistance to targeted therapy and immunotherapy. In addition, these EVs can deliver drug resistance-promoting molecules to other myeloma cells or immune cells, making them an important mechanism for therapeutic failure in MM. These metabolic characteristics may provide a metabolic vulnerability that can be targeted for therapeutic intervention (94, 95).

Our study identified C3 *ASS1*+ plasma cells as a highly malignant subpopulation associated with RRMM, which may serve as a potential prognostic marker and therapeutic target. The functional verification experiments of *ASS1* further confirmed its key role in maintaining the malignant phenotype of MM cells. The knockdown of *ASS1* in RPMI 8226 and U266 cell lines significantly inhibited the short-term and long-term proliferation of MM cells, reduced the clonogenic capacity, and inhibited cell migration, while promoting cell apoptosis. In addition, if our proposed mechanistic link between the C3 *ASS1*+ plasma cell subpopulation and EV-mediated signaling exists, inhibiting *ASS1* may exert multiple therapeutic effects beyond directly suppressing intrinsic tumor growth. For example, it may also change the vesicle-mediated intercellular signaling, thereby mitigating the immunosuppressive BMME in RRMM. Currently, these are only associative assumptions and direct experimental research is needed to determine whether *ASS1* functionally regulates EV biogenesis or cargo loading in MM.

However, this study has several limitations. First, the sample size is relatively small, with only 12 bone marrow samples included. Larger cohort studies are needed to validate our findings. Second, the study did not include clinical data such as treatment response and survival outcomes, which limits the clinical relevance of our findings. Future studies should integrate clinical data to further explore the prognostic value of plasma cell subpopulations and their association with treatment response. In future investigations, artificial intelligence (AI) and machine learning approaches can be applied to build predictive models for MM progression and relapse based on the expression signatures of the C3 *ASS1*+ plasma cell subpopulation (99, 100). And future work will profile immune checkpoint molecules and immunotherapeutic targets in plasma cell subpopulations, to define unique immunotherapeutic signatures of *ASS1*+ plasma cells. Third, the downstream molecular mechanisms of *ASS1* regulating the malignant phenotype of MM cells need to be further explored, so as to clarify the regulatory role of ASS1 in MM drug resistance and metabolic reprogramming. In addition, the current functional experiments are only based on *in vitro* cell lines. Primary bone marrow plasma cells from patients and the *in vivo* functional verification of *ASS1* in animal models are needed to further confirm its role in tumorigenesis and progression.

Beyond intercellular communication, the unique molecular cargo of MM-derived EVs holds significant promise as liquid biopsy biomarkers (101, 102). The EV secretion profile of C3 *ASS1*+ plasma cells, given their distinct metabolic and transcriptional characteristics, may yield novel biomarker candidates worthy of future investigation. Future studies should investigate whether circulating EV cargo reflective of C3 *ASS1*+ cell activity can serve as dynamic biomarkers of RRMM progression and treatment response.

In conclusion, our study provides a comprehensive analysis of the cellular heterogeneity, developmental trajectories, cell-cell communication patterns, TF regulatory networks, and metabolic characteristics of MM using scRNA-seq. The identification of C3 *ASS1*+ plasma cells as a highly malignant subpopulation was also confirmed by the functional validation experiments. Given the enrichment of C3 *ASS1*+ plasma cell subpopulation in RRMM patients and the oxidative phosphorylation pathway, and abnormal EV-related factor expression, this study established an extended mechanism model: High ASS1 expression in C3 *ASS1*+ plasma cells activates metastasis-promoting and immune-suppressive programs, inducing soluble factors selectively incorporated into EVs. These functional EVs diffuse through BMME to reprogram recipient cells, facilitating immune escape and enhancing therapeutic resistance. While the role of EVs in this pathway requires direct verification, this study provides new insights into MM heterogeneity and progression, first linking high-risk C3 *ASS1*+ plasma cell subpopulation to EV-mediated tumor-stromal communication. Targeting *ASS1* or EV release may be a potential multi-pronged MM therapy, especially for RRMM patients, and EVs or cargos from this subpopulation may serve as novel biomarkers for MM progression and therapy resistance.

## Data Availability

The single-cell sequencing datasets generated and/or analyzed in this study are publicly accessible in GEO (GSE223060). The original contributions presented in the study are included in the article/**Supplementary Material**. Further inquiries can be directed to the corresponding authors.

## Supplementary material

Supplementary Figure 1 Validation of ASS1 knockdown efficiency. (A) qRT–PCR analysis of ASS1 mRNA expression in RPMI 8226 and U266 cells following transfection with si-Ctrl, si-ASS1#1, or si-ASS1#2, confirming effective gene silencing. Data are presented as mean ± SD from three independent experiments. **P < 0.01 versus si-Ctrl.
