# Correction: Single-cell profiling uncovers extracellular vesicle-associated malignant plasma cell subpopulations driving multiple myeloma progression

**DOI:** 10.3389/fimmu.2026.1946841

**Published:** 2026-08-06

**Authors:** Lu Fan, Han Zhao, Mengya Cong, Mengxiao Zhang, Tuerxunayi Rouzi, Beibei Xie, Jifei Dai, Weiying Bao

**Affiliations:** 1 Department of Hematology, Songjiang Hospital Affiliated to Shanghai Jiao Tong University School of Medicine, Shanghai, China; 2 Shanghai Jiao Tong University School of Medicine, Shanghai, China; 3 Department of Hematology, The Second Affiliated Hospital of Anhui Medical University, Hefei, China; 4 Department of Hematology, The First Affiliated Hospital of Anhui Medical University, Hefei, Anhui, China

**Keywords:** ASS1, extracellular vesicles, ICAM, MIF, multiple myeloma, plasma cell subpopulation, scRNA-seq, tumor microenvironment

There was a mistake in **Figure 3** as published. Due to an oversight, the earlier version of **Figure 3** was mistakenly retained. The corrected **Figure 3** and its caption appear below.

**Figure 3:**
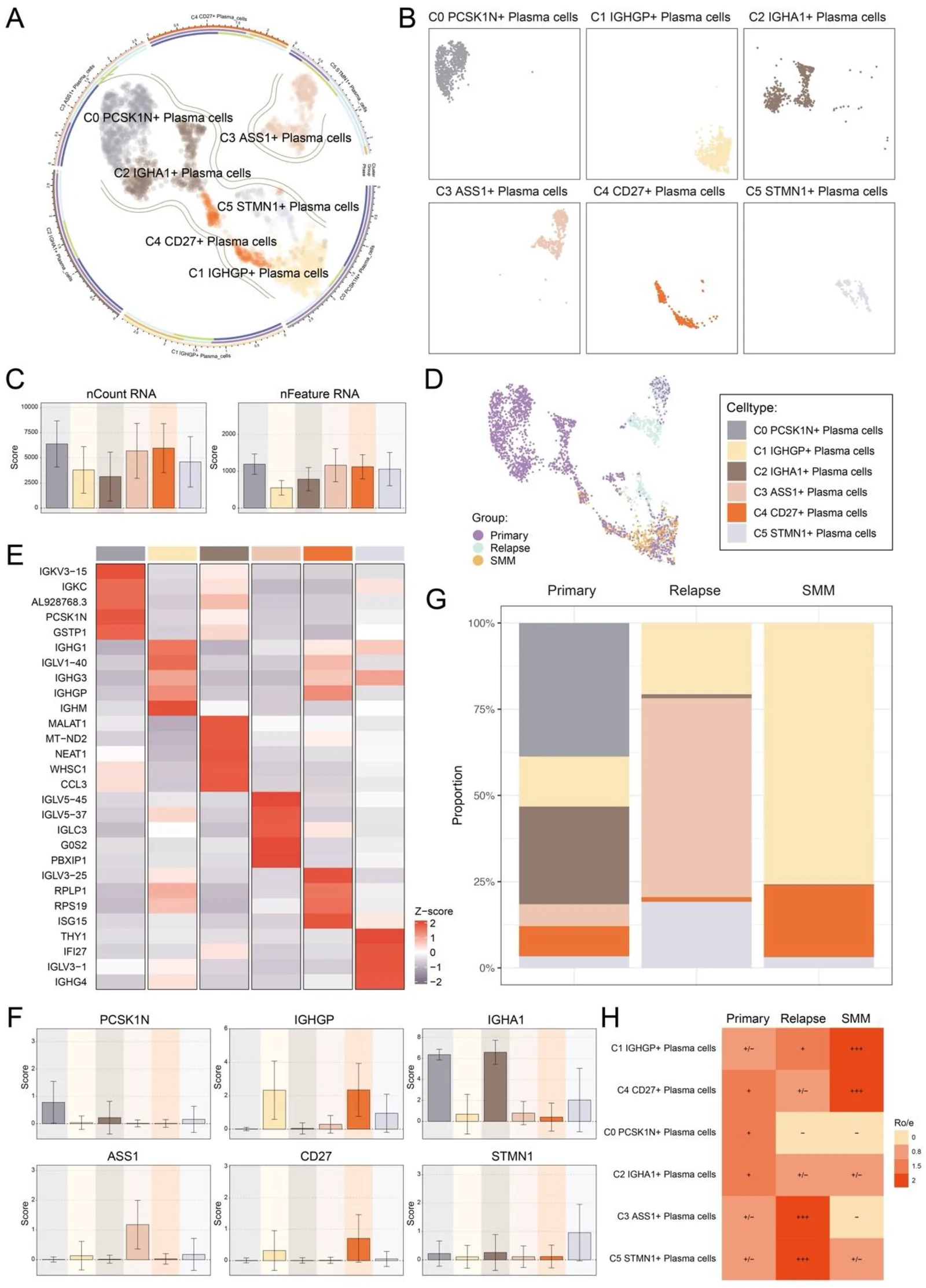
Heterogeneity of plasma cell subpopulations in SMM, NDMM, and RRMM. **(A)** UMAP plot showed 6 different plasma cell subpopulations. **(B)** Faceted UMAP plots illustrated the distribution characteristics of 6 plasma cell subpopulations, respectively. **(C)** Bar plots showed the nCount RNA and nFeature RNA scores of the 6 plasma cell subpopulations. **(D)** UMAP plot displayed the plasma cells colored according to disease subtypes. **(E)** Heatmap showed the expression of top 5 DEGs across the 6 plasma cell subpopulations. **(F)** Bar plots showed the expression of the named gene for each plasma cell subpopulation. **(G)** Stacked bar graphs illustrated the proportions of 6 plasma cell subpopulations. **(H)** Ro/e scores were used to assess the preferences of each cell type across different disease subtypes (SMM, NDMM, and RRMM). “Primary” refers to NDMM, while “Relapse” refers to RRMM.

The original article has been updated.

