# Supplementary figures and images for: Single-cell profiling uncovers extracellular vesicle-associated malignant plasma cell subpopulations driving multiple myeloma progression
Source: Front Immunol. 2026 Jul 15;17:1848792. doi: 10.3389/fimmu.2026.1848792 (PMC13416668; doi:10.3389/fimmu.2026.1848792)

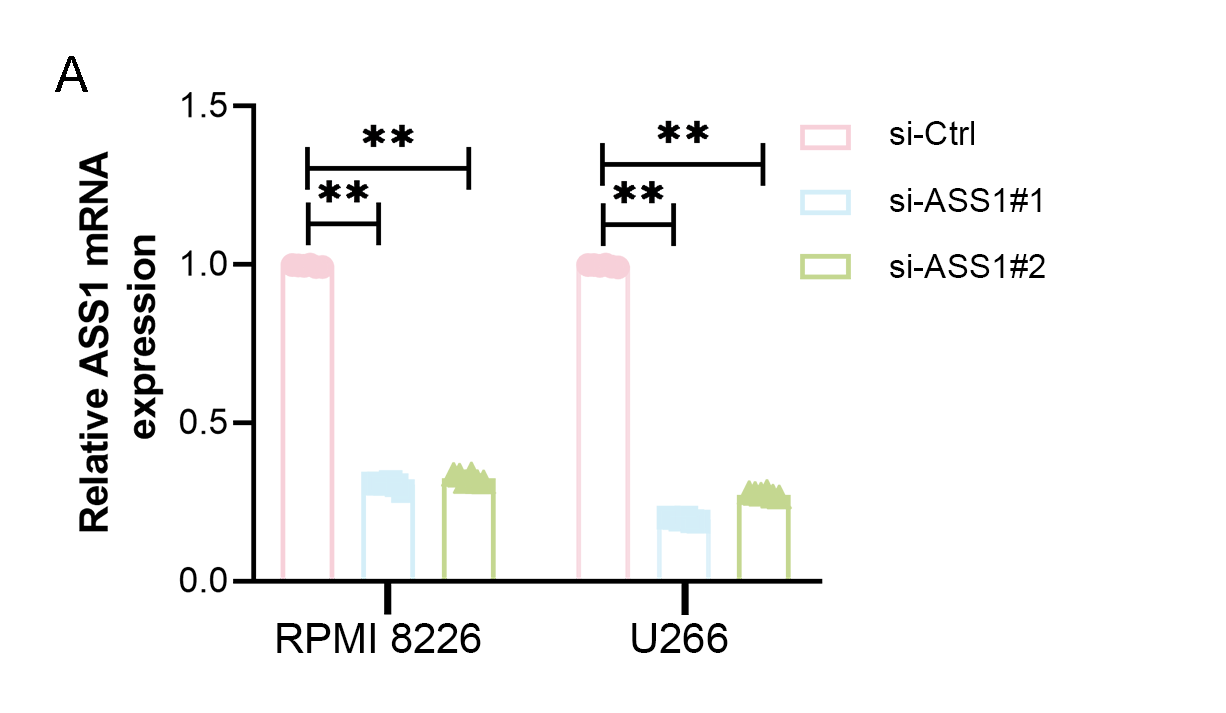

Supplement: Supplementary Figure 1 — Validation of ASS1 knockdown efficiency. (A) qRT–PCR analysis of ASS1 mRNA expression in RPMI 8226 and U266 cells following transfection with si-Ctrl, si-ASS1#1, or si-ASS1#2, confirming effective gene silencing. Data are presented as mean ± SD from three independent experiments. **P < 0.01 versus si-Ctrl. [file Image1.tif]
