# Supplementary material for: Single-cell profiling uncovers extracellular vesicle-associated malignant plasma cell subpopulations driving multiple myeloma progression
Source: Front Immunol. 2026 Jul 15;17:1848792. doi: 10.3389/fimmu.2026.1848792 (PMC13416668; doi:10.3389/fimmu.2026.1848792)
Supplement: Supplementary file 2 [file Table1.docx]

| **Target gene** | **Name** | **Sequence (5′→3′)** | **Application** | **Supplier** |
| --- | --- | --- | --- | --- |
| ASS1 | si-ASS1#1 (sense) | GCUCUACAGUGAUGAAGAUTT | siRNA knockdown | GenePharma (Shanghai, China) |
|  | si-ASS1#1 (antisense) | AUCUUCAUCACUGUAGAGCTT |  |  |
| ASS1 | si-ASS1#2 (sense) | GCCAGAAUGUGAAGCUAAATT | siRNA knockdown | GenePharma (Shanghai, China) |
|  | si-ASS1#2 (antisense) | UUUAGCUUCACAUUCUGGCTT |  |  |
| Negative control | si-Ctrl (sense) | UUCUCCGAACGUGUCACGUTT | siRNA control | GenePharma |
|  | si-Ctrl (antisense) | ACGUGACACGUUCGGAGAATT |  |  |
| ASS1 | Forward primer | CAGGAGGACCTGGTGAAGAA | qRT–PCR | Sangon Biotech (Shanghai, China) |
|  | Reverse primer | TGGTAGTTGGTGATGGCAGT |  |  |
| GAPDH | Forward primer | GGAGCGAGATCCCTCCAAAAT | qRT–PCR | Sangon Biotech |
|  | Reverse primer | GGCTGTTGTCATACTTCTCATGG |  |  |

**Supplementary Table 1. Sequences of siRNAs and qRT–PCR primers used in this study**
